# Mechanical and morphological properties of the aortic root and arch late after arterial switch operation for transposition of the great arteries

**DOI:** 10.1186/1532-429X-14-S1-P115

**Published:** 2012-02-01

**Authors:** Hopewell Ntsinjana, Giovanni Biglino, Jennifer A Steeden, Silvia Schievano, Andrew M Taylor

**Affiliations:** 1 Centre for Cardiovascular Imaging, UCL Institute of Cardiovascular Science, London, UK

## Background

The arterial Switch operation (ASO) is now performed routinely for the repair of transposition of the great arteries (TGA). Neo-aortic root dilatation and reduced elasticity of the ascending aorta have been shown to impact the long-term outcomes of these patients.

The aim of this study was to assess the effect of the ASO with the Lecompte maneuver on the dynamic behavior and dimensions of the aortic root, and the shape and curvature of the ascending aorta and aortic arch.

## Methods

Data was reviewed from nineteen TGA subjects (14.3±2.6 years, 1.6±0.3 m2 BSA) and twenty matched control subjects (15.0±2.4 years, 1.7±0.2 m2 BSA), who had undergone cardiovascular magnetic resonance (CMR) imaging for clinical indications at our Institution. Aortic flows at the root level and 3D Whole-Heart bSSFP were obtained using standard CMR sequences. Both groups had non-invasive brachial blood pressure cuff measurements. 3D Whole-Heart bSSFP images were used to create 3D models for each patient using commercial software (Mimics, Materialise, Belgium) and the volume 3D centerline from ventricular apex to the descending aorta was calculated in order to better describe aortic geometry. Aortic flows and area changes were then analyzed from magnitude and phase-contrast images with an in-house written plugin (Osirix, Pigmeo, Switzerland) using an automatic propagation algorithm based on non-rigid registration.

## Results

**Figure 1:**
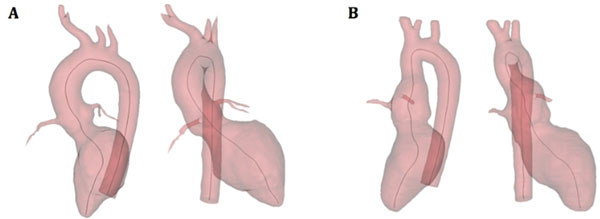
Two views of 3D volume centerline from ventricular apex to the descending aorta, showing the different curvature smoothness and angulation between the control case (A) and the arterial switch case (B). Curvature (= 1/radius at highest point of the transverse aorta) was 0.047 mm^-1 for the control case (A) and 0.140 mm^-1 for the ASO case (B).

**Figure 2:**
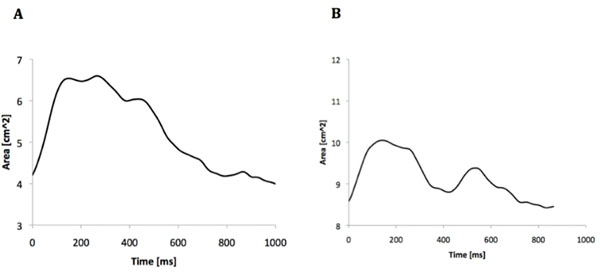
Change in area for the control case (A) and ASO case (B). Note the 'double-peak' curve in the ASO case.

ASO subjects showed an abnormal aorta from the root to the arch with dilated aortic root and a more fragmented curvature of the ascending aorta with acute angulation of the aortic arch (Figure 1). Dilatation of the aortic root was confirmed by area measurements, with ASO subjects having significantly larger root area (7.3±2.4 vs 3.5±0.8 cm2, p<0.0001). Since no significant difference in pressure pulse was measured between the two groups (53.2 vs 53.4 mmHg, p=NS), % area change was taken as an indication of distensibility, with the ASO group exhibiting decreased values (23.3±10.4 vs 52.4±9.9 %, p<0.0001). Finally, ASO patients also showed an abnormal dynamic area change behavior, with a double-peak area curve rather than an aortic pressure-looking area curve (Figure 2B).

## Conclusions

Changes in morphology, dimensions and dynamics observed in the ASO group suggest overall increased impedance, which is likely to be the mechanistic explanation for the long-term outcomes of this procedure.

## Funding

Carnegie Foundation, Fondation Leducq, UK National Institute of Health Research (NIHR), Royal Academy of Engineering and EPSRC.

